# Neutral hip position for the oblique lumbar interbody fusion (OLIF) approach increases the retroperitoneal oblique corridor

**DOI:** 10.1186/s12891-020-03592-9

**Published:** 2020-08-31

**Authors:** Vit Kotheeranurak, Weerasak Singhatanadgige, Chindarat Ratanakornphan, Wicharn Yingsakmongkol, Richard A. Hynes, Worawat Limthongkul

**Affiliations:** 1 Department of Orthopedics, Queen Savang Vadhana Memorial Hospital, Chonburi, Sriracha Thailand; 2 Department of Orthopaedics, Faculty of Medicine, Chulalongkorn University, 1873 Rama IV Rd, Pathumwan, Bangkok, 10330 Thailand; 3 Radiology Department, King Chulalongkorn Memorial Hospital, Bangkok, Thailand; 4 The B.A.C.K. Center, Florida, Melbourne USA

**Keywords:** Retroperitoneal oblique corridor, ROC, Oblique lateral lumbar interbody fusion, OLIF, Hip position, MRI

## Abstract

**Background:**

The prepsoas lateral approach for spinal fusion, oblique lateral lumbar interbody fusion (OLIF), is considered one of the minimally invasive spinal fusion methods and is gaining popularity due to improved outcomes with copious supporting evidence. To date, no publication has studied the various positions of the left hip in actual patients which might affect the retroperitoneal oblique corridor (ROC). The study aimed to find the relevancy of the left hip position and the size of ROC.

**Methods:**

We recruited 40 consecutive patients who needed diagnostic MRI from the out-patient clinic. MRI scan from L2 to L5 was performed in the supine, right lateral decubitus with hip flexion, and right lateral decubitus with hip in a neutral position. The retroperitoneal oblique corridor (ROC) was measured at the intervertebral disc level and compared.

**Results:**

ROC of the hip in neutral position was significantly larger than hip flexion in all levels (*p* < 0.05); there was no significant difference in the ROC among levels (*p* = 0.22). ROC seems to be largest at L2/3 followed by L3/4 and L4/5 respectively in all positions.

**Conclusions:**

The retroperitoneal oblique corridors of L2 to L5 were significantly increased when the hip is in the neutral position, while the psoas cross-sectional area and anterior thickness were minimized in this position. Surgeons might benefit from a neutral position of the left hip in the oblique lateral lumbar interbody fusion (OLIF) procedure.

In conclusion, the retroperitoneal oblique corridors of L2 to L5 were significantly increased when the hip is in the neutral position, while the psoas cross-sectional area and anterior thickness were minimized in this position. Surgeons might benefit from a neutral position of the left hip in the oblique lateral lumbar interbody fusion procedure.

## Background

Spinal fusion via the lateral approach is considered as one of the minimally invasive spinal fusion procedures and is gaining more popularity owing to its good outcomes. Since Mayer [1] published the minimally invasive access via the anterior approach in the literature, many have developed the technique and described the pre-psoas approach known as oblique lumbar interbody fusion (OLIF), which was later popularized by many surgeons [2–6].

Preoperatively, the lumbar spine MRI images in the supine position are used to determine the presence of the retroperitoneal oblique corridor (ROC), located between the aorta or the left common iliac artery and the left psoas muscle, to ensure safety and reduce complications during the surgery [7–10]. This ROC passage allows the surgeon to perform the intervertebral disc preparation without having to go through the psoas muscle and thereby risking injury to the lumbar plexus.

However, Zhang et al. [11] showed that the ROC would become even narrower from the supine to the right lateral decubitus, implying that the use of ROC from a supine position will not be perfectly accurate. To date, there is no strict rule on how the left hip should be positioned and no publication has studied the various positions of the left hip which might affect the ROC. The objective was to find the relevancy of the left hip position and the size of ROC. We hypothesized that the ROC would be affected by the position of the left hip in the right lateral decubitus position.

## Methods

This study is a prospective cross-sectional study which followed the STROBE guidelines. Forty consecutive patients who needed diagnostic lumbosacral MRI were enrolled. We excluded patients with any previous surgery that affected the psoas muscle and whenever there was a contraindication to perform MRI. Patients who had no ROC, diminished in the potential space between the aorta and psoas muscle, were excluded due to the inability to measure the ROC dimension.

**Fig. 1:**
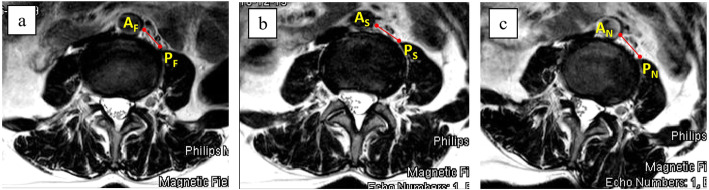
The retroperitoneal oblique corridor (ROC) measured in the three different positions; supine position: ROC_S_ **a**, the right lateral decubitus with the left hip in flexion: ROC_HF_ **b**, and the right lateral decubitus with the left hip neutral: ROC_HN_ **c**. Noted the differences in left psoas morphologies and thickness

**Fig. 2:**
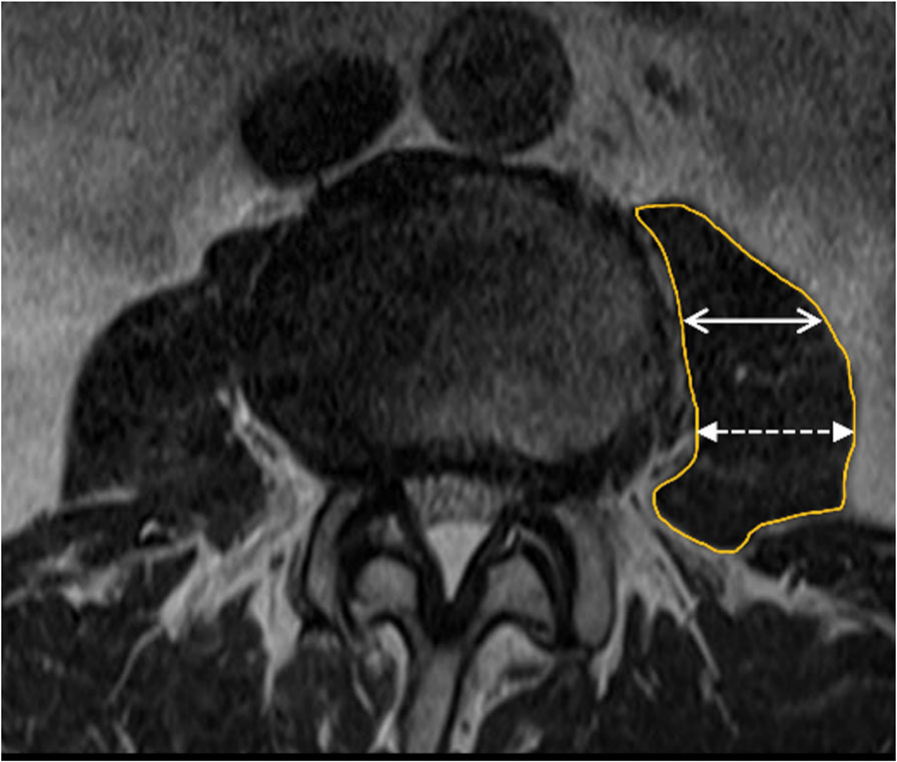
Demonstration of manually delineated of the left psoas cross-sectional area (line), the anterior 1/3 of the left psoas muscle (two-headed arrow) and the posterior 1/3 of the left psoas muscle (dotted two-headed arrow)

Three sequences of MRI (3 T, Ingenia, Phillips, USA, 2 mm cut) were done as follows: T1 and T2 sequences supine (with knees slightly flexed), T2 sequence in the right lateral decubitus with the left hip in a neutral position, and T2 sequence in the right lateral decubitus with the left hip in 45 degree-flexion. All images were then measured in the axial plane using the Picture Archiving and Communications System (PACS) by a spine surgeon and a radiologist. Measured at the mid-intervertebral disc height, we described the ROC at each level as the distance between A and B, where A was the left-most lateral border of the aorta (or the left iliac artery), and B was the most anteromedial aspect of the left psoas muscle (Fig. 1). Distances AB (ROC) were measured at each intervertebral disc level from L2/3 to L4/5 in all of the three sequences: the supine (ROC_S_), the right lateral decubitus with hip in neutral (ROC_HN_), and the right lateral decubitus with the hip flexed 45 degrees (ROC_HF_). The left psoas morphologies were manually delineated and calculated for the cross-sectional areas (PCA) after dividing the psoas muscle into 3 sections equally. The thickness of the anterior (APT) 1/3 of the psoas muscle was measured for comparisons (Fig. 2).

Statistical analysis was performed using the SPSS v23.0 software (IBM SPSS Statistics for Windows, IBM Corp., Armonk, NY). The data were expressed as the mean and standard deviation (SD). An ANOVA with repeated measures was used to compare the ROC, PCA, and the psoas thickness at each level in each position. The interobserver reliability was examined using Pearson’s correlation coefficient of the first and second measurements. A biostatistician performed the analysis.

## Results

The mean age of the patients was 45.6 ± 2.7 years (44–65). Of the 40 patients, 27 were female and 13 were male. Their BMI was 16.5–24.3 (22.3 ± 1.5) kg/m^2^. At L2/3, the largest ROC was found at ROC_HN_ (16.8 ± 1.4 mm) as compared to ROC_S_ (14.8 ± 1.3 mm) and ROC_HF_ (13.0 ± 0.9 mm) (*p* = 0.021). At L3/4, the largest ROC was found at ROC_HN_ (16.2 ± 0.9 mm) as compared to ROC_S_ (14.3 ± 2.2 mm) (*p* = 0.036) and ROC_HF_ (13.1 ± 1.3 mm). At L4/5, the largest ROC was found at ROC_N_ (15.4 ± 1.0 mm) as compared to ROC_S_ (13.5 ± 1.3 mm) and ROC_HF_ (12.8 ± 1.8 mm) (*p* = 0.019), as shown in Table 1.

**Table 1:** Retroperitoneal oblique corridor (ROC) according to lumbar levels (mm)

	ROC_S_	ROC_HF_	ROC_HN_	*p* value
L2/3	14.8 ± 1.3	13.0 ± 0.9	16.8 ± 1.4	0.021*
L3/4	14.3 ± 2.2	13.1 ± 1.3	16.2 ± 0.9	0.036*
L4/5	13.5 ± 1.3	12.8 ± 1.8	15.4 ± 1.0	0.019*

* indicates *p < 0.05, statistical significance*

*ROC*_*S*_ retroperitoneal oblique corridor in supine

*ROC*_*HF*_ retroperitoneal oblique corridor in right lateral decubitus with hip flexion

*ROC*_*HN*_ retroperitoneal oblique corridor in right lateral decubitus with hip in neutral

There were statistical differences between ROC_HN_ compared to the ROC_S_, and ROC_HF_ at all levels (*p* < 0.05) but no significant difference of ROC between all levels (*p* = 0.22).

The relative psoas cross-sectional area was significantly smaller (*p* < 0.05) in the right lateral decubitus with the hip in the neutral position at all levels (L2/3 = 530.6 ± 32.1 mm^2^, L3/4 = 870.9 ± 21.9 mm^2^, and L4/5 = 1065.6 ± 42.2 mm^2^) when compared to the supine position (L2/3 = 572.6 ± 28.9 mm^2^, L3/4 = 917.3 ± 34.2 mm^2^, and L4/5 = 1180.9 ± 33.7 mm^2^) or the right lateral decubitus and hip in flexion (L2/3 = 584.8 ± 42.6 mm^2^, L3/4 = 964.6 ± 32.5 mm^2^, and L4/5 = 1123.2 ± 22.7 mm^2^), as shown in Table 2.

**Table 2:** Relative psoas cross-sectional area (PCA) according to lumbar levels (mm^2^)

	PCA_S_	PCA_HF_	PCA_HN_
L2/3	572.6 ± 28.9	584.8 ± 42.6	530.6 ± 32.1*
L3/4	917.3 ± 34.2	964.6 ± 32.5	870.9 ± 21.9*
L4/5	1180.9 ± 33.7	1123.2 ± 22.7	1065.6 ± 42.2*

* indicates *p < 0.05*

*PCA*_*S*_ relative psoas cross-sectional area in supine

*PCA*_*HF*_ relative psoas cross-sectional area in right lateral decubitus with hip flexion

*PCA*_*HN*_ relative psoas cross-sectional area in right lateral decubitus with hip in neutral

There were statistical differences between PCA_HN_ compared to the PCA_S_ and PCA_HF_ at all levels (*p* < 0.05).

The morphological changes of the psoas muscle in the neutral hip position resulted in the statistically (*p* < 0.05) smallest of the anterior 1/3 of the psoas thickness (APT) at L3/4 and L4/5 levels (L2/3 = 13.6 ± 4.3 mm, L3/4 = 20.2 ± 5.9 mm, and L4/5 = 28.4 ± 2.7 mm) as compared to the supine position (L2/3 = 14.9 ± 2.1 mm, L3/4 = 23.2 ± 4.8 mm, and L4/5 = 30.9 ± 7.2 mm) and the right lateral decubitus and hip in flexion (L2/3 = 15.9 ± 3.7 mm, L3/4 = 23.8 ± 4.9 mm, and L4/5 = 32.2 ± 2.1 mm), as shown in Table 3. The interobserver agreement was at an almost-perfect level (kappa = 0.83).

**Table 3:** Anterior 1/3 of psoas thickness (APT) according to lumbar levels (mm)

	APT_S_	APT_HF_	APT_HN_
L2/3	14.9 ± 2.1	15.9 ± 3.7	13.6 ± 4.3
L3/4	23.2 ± 4.8	23.8 ± 4.9	20.2 ± 5.9*
L4/5	30.9 ± 7.2	32.2 ± 2.1	28.4 ± 2.7*

* indicates *p < 0.05*

*APT*_*S*_ anterior 1/3 of psoas thickness in supine

*APT*_*HF*_ anterior 1/3 of psoas thickness in right lateral decubitus with hip flexion

*APT*_*HN*_ anterior 1/3 of psoas thickness in right lateral decubitus with hip in neutral

There were statistical differences between APT_HN_ compared to the APT_S_ and APT_HF_ at L3/4 and L4/5 (*p* < 0.05).

## Discussion

The retroperitoneal oblique corridor is one of the prerequisites for OLIF surgery; thus, many surgeons have sought to find a method to maximize it. The ROC should be assessed during the preoperative planning of the OLIF procedure. In recent years, a few publications have studied the ROC for the oblique approach. Davis et al. [12] investigated the ROC in 20 cadavers in the lateral position without tilting the table and showed a potential corridor between the belly of the psoas and the vessel of L2-S1. They also found that the ROC would increase up to 59% with mild psoas retraction. Molinares et al. [13] demonstrated by the use of 133 MRIs to assess the ROC preoperatively that the ROCs were presented most in L3/4 and L2/3 followed by L4/5 and L5/S1. Their cornerstone study has encouraged the use of MRI as a preoperative tool to determine the presence of the ROC for oblique access to the intervertebral disc space. Similar to our study, they found that the corridor was widest at the L2/3 disc level.

Although the ROC should be determined before OLIF surgery, the ROC measured in routine supine MRI may not reflect the true ROC during surgery. Zhang et al. [11] compared the ROC of L1–5 using MRI between the supine and right lateral decubitus position and found that the ROC was narrower in the latter position. However, the position of the hip had not been determined in their study. Our result confirmed the results of the Zhang study that the ROC was narrower in the right lateral decubitus with mildly hip flexion as compared to the supine position. The mean ROC value from our study was ranged from 12.8–13.0 mm compared to 8.6–15.5 mm from their study). Furthermore, we demonstrated that the hip position significantly affects the ROC. With the hip in a neutral position, the ROC is widest when compared to the hip flexion in the lateral or supine positions. Even the significant value was few millimeters, this insight is clinically valuable for supporting the proper position of the left hip in order to maximize the retroperitoneal corridor dimension up to 20% as compared to average ROC in several studies (12.3–15.5 mm) ( [11–13]). However, flexion of the hip is not desirable to increase the ROC [2, 3, 14].

Although not having gone through the psoas muscle, which contains neural structures, there are reports of postoperative numbness, pain, and other neurologic complications [7, 8]. This could be best explained by the retraction or stretch of the psoas muscle itself. In other words: the less psoas retraction, the less neurological sequelae [7]. The concept might be different from the transpsoas approach which nerve relaxation is thought to be safer when the hip is in slight flexion. Also, it is shown from the tractography study that when the hip is flexion, the lumbar plexus tends to move posteriorly which offers a safer passage for the transpsoas procedure [15].

From our results, which are consistent with previous studies by Molinares and Zhang [11, 13], the ROC was narrowest at the L4/5 level. This implies that L4/5 is more susceptible to psoas retraction; therefore, care should be taken when performing the retraction to the psoas muscle, especially in the lower lumbar levels. The genitofemoral nerve is commonly encountered on the anterior psoas at the L4/5 level and special care should be taken no to over retract or injure it during psoas manipulation.

According to our results, the possible biomechanical explanation could be a result of the psoas muscle tension and its morphological difference between the hip positions. When the hip is flexed, the psoas muscle tends to be shortened and relaxed. When the hip is in a neutral position, it tends to be stiffened by tension, gaining more passive tension at the same time and as a result, the ROC is increased [16]. As shown in our study, the psoas muscle gets smaller in the cross-sectional area when the hip position changes to neutral, as supported by the smallest PCA in the neutral position. The other issue is that the thickness of psoas muscle changing its morphology can be beneficial in the transpsoas approach. Psoas muscle fiber relaxation by flexion of the hip, intuitively, would splay the fibers more from anterior to posterior, as this relaxes the tension by shortening the distance of the lesser trochanteric insertion to the insertions under the outer third of the transverse process. The reason we did not measure the AP diameter of the psoas muscle because of its difficulty and unreliable reference point from the changes in psoas morphology. And lastly, the gravitational mobilization of the Aorta may be one of the factors contributing to the widening of the ROC.

Lumbar lordosis is another concern when we perform the OLIF. In order to achieve the optimal balance both locally and globally, it more straightforward to gain lumbar lordosis prior to the interbody insertion. The hip flexion, undoubtedly, decreases the lumbar lordosis. On the contrary, a neutral position of the hip would maximize lumbar lordosis which opens the anterior disc spaces that facilitate surgery during intervertebral disc entry. This could be useful when we perform an L5/S1 OLIF surgery: the hip needs to be neutral, not flexed in order to approach the angle of the high lordosis of the L5/S1 disc.

The limitations of this study are that the number of patients was limited and the exclusivity of the Asian population in the sample. L5/S1 was not included in the study due to the variation of left common iliac artery and vein, which may affect the measurement.

## Conclusions

In conclusion, the retroperitoneal oblique corridors of L2 to L5 were significantly increased when the hip is in the neutral position, while the psoas cross-sectional area and anterior thickness were minimized in this position. Surgeons might benefit from a neutral position of the left hip in the oblique lateral lumbar interbody fusion (OLIF) procedure.

## Data Availability

The datasets used and/or analysed during the current study are available from the corresponding author on reasonable request.

## Abbreviations

OLIF: Oblique lateral lumbar interbody fusion
ROC: Retroperitoneal oblique corridor
PCA: Psoas cross-sectional area
APT: Anterior psoas thickness
SD: Standard deviation
